# Violet Light Down-Regulates the Expression of Specific Differentiation Markers through Rhodopsin in Normal Human Epidermal Keratinocytes

**DOI:** 10.1371/journal.pone.0073678

**Published:** 2013-09-17

**Authors:** Hyoung-June Kim, Eui Dong Son, Ji-Yong Jung, Hyun Choi, Tae Ryong Lee, Dong Wook Shin

**Affiliations:** 1 Bioscience Research Institute, Amorepacific Corporation R&D Center, Yongin-city, Gyeonggi-do, Republic of Korea; 2 Department of Biological Sciences, Sungkyunkwan University, Suwon-city, Republic of Korea; University of Tennessee, United States of America

## Abstract

Several recent reports have demonstrated that photoreceptors are expressed in human skin. The rod and cone photoreceptor-like proteins are expressed in human skin and rhodopsin, long wavelength-opsin, and short wavelength-opsin are also present in cultured murine melanocytes. Furthermore, the photopigment rhodopsin is expressed in human melanocytes and is involved in ultraviolet A phototransduction which induces early melanin synthesis. In this study, we investigated whether rhodopsin is expressed and plays any physiological roles in the normal human epidermal keratinocytes (NHEKs). We found that rhodopsin was expressed and localized on the plasma membrane in NHEKs, and only violet light among several wavelengths within the visible range significantly increased the expression of rhodopsin mRNA. We further found that rhodopsin over-expression decreased the mRNA expression levels of keratinocyte differentiation markers, such as keratin-1 and keratin-10, and violet light also decreased the mRNA expression levels of keratinocyte differentiation markers and these decreased expression levels were recovered by a rhodopsin-directed siRNA. Moreover, we further demonstrated that violet light significantly decreased the phosphorylation levels of cAMP responsive element-binding protein (CREB) and that it more effectively decreased the phosphorylation of CREB when rhodopsin was over-expressed. In addition, we observed that pertussis toxin, a Gα_i_ protein inhibitor, restored the rhodopsin-induced decrease in the differentiation markers in NHEKs. Taken together, these results suggest that rhodopsin down-regulates the expression levels of specific keratinocyte differentiation markers via the Gα_i_ signaling pathway in NHEKs.

## Introduction

The skin acts as a barrier against various environmental factors, such as pathogens, chemical and physical stimuli, and light exposure. It has the capability not only to recognize, but also count these signals and integrate into organized responses [1], [2]. In particular, the skin is continuously exposed to a spectrum of solar radiation, which mostly consists of ultraviolet (UV), visible, and infrared light. UV radiation causes skin disorders, for example, by increasing keratinocyte proliferation and inducing epidermal hyperplasia [3], [4]. It has been reported that acute irradiation with UVB concomitantly increases epidermal proliferation and the expression of differentiation markers *in vivo* [4]. Chronic UVA irradiation at low levels also induces epidermal hyperplasia and increases the thickness of the stratum corneum in human skin [5]. It is also well-known that melanocytes shows melanogenic activity and complex neuroendocrine activity in response to ultraviolet radiation [6]–[8].

Unlike UV radiation, little is known regarding the effect of other wavelengths, such as visible light (380–780 nm), on human skin. Recent reports have demonstrated that blue light exposure reduced the proliferation and induced the differentiation of keratinocytes [9]. In addition, red light exposure increased the proliferation of keratinocytes [10] and fibroblasts [11]. Interestingly, it has also been shown that visible light can affect skin barrier recovery in hairless mice. For examples, red light (550–670 nm) can accelerate the recovery of a disrupted skin barrier, whereas blue light (430–510 nm) delays its recovery [12].

According to previous reports, light-induced biological responses require chromophores (photoacceptors), which are converted into an excited state by absorbing the light and then act on cellular targets [13], [14]. For examples, 7-dehydrocholesterol (7-DHC) absorbs UVB radiation in the skin and is transformed to previtamin D_3_, which is eventually isomerized to vitamin D_3_ in a temperature-dependent manner [15], [16]. Cytochrome c oxidase in visible-to-near IR light and porphyrin-containing enzymes and flavoproteins in blue light are good examples of photoreceptors that contain chromophores [17]–[20]. Thus, there might be photoreceptors that elicit visible light phototransduction in the skin. Indeed, a few studies have recently demonstrated that rod and cone photoreceptor-like proteins are expressed in human skin [21] and that rhodopsin, long wavelength (LW)-opsin, and short wavelength (SW)-opsin are present in cultured murine melanocytes [22]. Moreover, RPE65, an isomerase that converts all-trans-retinal to 11-cis-retinal in the retinal pigment epithelium [23], [24], is also expressed in human keratinocytes [25]. A recent study reported that the photopigment rhodopsin is expressed in human epidermal melanocytes (HEMs) and is involved in UVA phototransduction, which rapidly induces Ca^2+^ mobilization and early Ca^2+^-dependent melanin synthesis [26].

Rhodopsin is a well-known G protein-coupled receptor expressed in retinal rod cells and is located in the disk and plasma membranes of the retinal rod outer segment [27]. Rhodopsin can detect photons and transfers signals to other neuronal cells in the retina [28]. In visual signal transduction, rhodopsin detects a broad visible spectrum of wavelengths between 380 and 543 nm (λ max ∼500 nm) with a photoisomerization of 11-cis- to all-trans-retinal chromophores. After photoisomerization, metarhodopsin-II (the activated form of rhodopsin) absorbs at approximately 380 nm and can catalyze the guanine nucleotide exchange by the rod cell G protein, transducin (G_t_) [29].

Although it is known that rhodopsin and other opsins are expressed in human skin, particularly in the epidermis, which consists mainly of keratinocytes and melanocytes [21], [26], the biological roles and signaling pathways of the photoreceptors in human skin, as well as their specific action spectra, remain to be elucidated.

In this study, we investigated whether rhodopsin plays any physiological roles in the normal human epidermal keratinocytes (NHEKs). We found that violet light specifically increased the mRNA level of rhodopsin and decreased the mRNA expression levels of specific keratinocyte differentiation markers in NHEKs. We further showed that the biological activity of violet light on the expression of specific differentiation markers was mediated by rhodopsin through down-regulation of CREB phosphorylation in NHEKs.

## Materials and Methods

### Cell Culture

Normal human epidermal keratinocytes (NHEKs) from neonatal foreskin were purchased from Lonza (Basel, Switzerland) and cultured in KBM-GOLD medium with KGM-GOLD growth supplements containing insulin, human epidermal growth factor, bovine pituitary extract, hydrocortisone, epinephrine, transferrin, and gentamicin/amphotericin B. The cells were serially passaged at 70–80% confluence, and the experiments were performed using subconfluent cells within two passages when the cells were actively proliferating.

### LED Irradiation System and UVA Source

**Figure 1:**
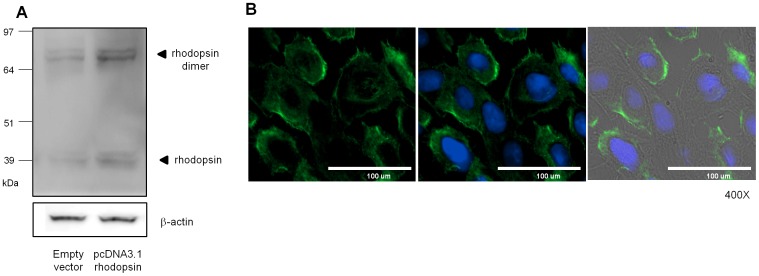
Rhodopsin was expressed in NHEKs. (A) NHEKs were transiently transfected with pcDNA3.1 empty vector (left lane) or rhodopsin expression vector (right lane) for 48 hr and then analyzed by Western blot. (B) The left panel represents the localization of endogenous rhodopsin (green) at the plasma membrane; the middle panel represents an overlay of rhodopsin and the nucleus stained with DAPI (blue); and the right panel represents an overlay of the middle panel and the phase contrast image.

The LED irradiation system, which emits light with a narrow bandwidth inside a CO_2_ incubator, was purchased from HanaroTR (Suwon, Korea). Internal LED arrays, which emit violet light (380–420 nm, λ_max_: 410 nm), green light (450–560 nm, λ_max_: 505 nm), orange light (560–620 nm, λ_max_: 590 nm) and red light (620–690 nm, λ_max_: 660 nm), could be controlled from 1 to 60 mW/cm^2^ when the object was 50 mm from the LED arrays. These arrays were obtained from Roithner Lasertechnik GmbH (Wien, Austria). This LED irradiation system emits narrower bandwidths than those provided by other commercial LED systems (Figure 1). We also used a spectroradiometer SPR-4001 (Luzchem, Ottawa, Canada) to determine the intensity of the irradiation. For UVA irradiation, we used a Biosun UV irradiation system with a lamp that produces wavelengths at approximately 350–390 nm (λ_max_: 365 nm) (Vilber Lourmat, Marnes-la-Valle-e, France). This system has a tray for the culture dishes, and the temperature did not exceed 30°C during exposure.

### Light Exposure and Treatment Using Various Inhibitors

Prior to irradiation, the cells were washed twice with PBS and irradiated in PBS with the indicated exposure lights to avoid the side effects associated with photosensitization in the culture medium. To avoid any confounding effects from other factors (temperature, humidity and CO_2_), the LED arrays were placed inside the CO_2_ cell culture incubator during exposure. The cells were then irradiated with UVA 1 and 10 J/cm^2^ (5–6 mW/cm^2^ for 3 and 30 min, respectively) or LED lights at 10 or 50 J/cm^2^ (27.8 mW/cm^2^ for 6 and 30 min, respectively). For the inhibition experiments, the NHEKs were pre-incubated for 1 hr with 50 ng/ml pertussis toxin (Sigma, St. Louis, MO, USA). After pre-incubation with pertussis toxin, the cells were irradiated with violet light at 50 J/cm^2^ (27.8 mW/cm^2^, 30 min). 24 hr after irradiation, Q-RT-PCR was performed and Western blot analyses were performed at 10 or 60 min after irradiation. In addition, 10 mM BAPTA/AM (Calbiochem, La Jolla, CA, USA) and 40 mM N-(6-amino-hexyl)-5-chloro-1-naphthalensulfonamide (W7) (Calbiochem) were used as a Ca^2+^ chelator and calmodulin antagonist, respectively. After pre-incubation with BAPTA/AM or W7, the cells were irradiated with violet light at 50 J/cm^2^ (27.8 mW/cm^2^, 30 min). 24 hr after irradiation, Q-RT-PCR was performed.

### Cell Viability Tests

NHEK cell viability after light exposure or transfection was evaluated using the WST-1 assay according to the manufacturer’s instructions (Roche Molecular Biochemical, Indianapolis, IN, USA). The cell viability detecting reagent 4-3-[4-lodophenyl]-2-4(4-nitrophenyl)-2H-5-tetrazolio-1,3-benzene disulfonate (WST-1; 10 mM pure solution) was added to the NHEKs in culture, and the cells were incubated for 2 hr in a humidified atmosphere. The absorbance at 450 nm was then measured, and the cell viability was expressed as the percentage of the absolute optical density of each sample relative to that of the control value.

### Over-expression or Knockdown of Rhodopsin

For the rhodopsin over-expression studies, we used an empty pcDNA3.1 vector or pEGFP-N1 plasmid, which encodes human rhodopsin. Briefly, NHEKs were seeded in 60 mm dishes for 24 hr and then transfected for 48 hr with 1 µg/ml of DNA using the X-tremeGENE HP DNA transfection reagent (Roche Diagnostics GmbH, Mannheim, Germany). To knockdown rhodopsin, the NHEKs were transfected for 24 hr with 50 nM of ON-targetplus SMARTPOOL siRNA (Non-targeting#2, #D-001810-02 and human rhodopsin, #L-005722-01-0005 and L-005722-00-0020), according to the manufacturer’s instructions (Thermo Fisher Scientific, Lafayette, CO, USA). The NHEKs were seeded in 60 mm dishes 24 hr prior to transfection and then transfected with siRNA using RNAiMAX (Invitrogen, Carlsbad, CA, USA) and OPTI-MEM for 12 hr. The medium was then changed to KGM-GOLD containing all of the appropriate supplements.

### RNA Extraction and Quantitative Real-time RT-PCR (Q-RT-PCR)

Total RNA was isolated using TRIzol™ (Invitrogen, Carlsbad, CA, USA), according to the manufacturer’s instructions. The RNA concentration was determined spectrophotometrically, and the integrity of the RNA was assessed using a BioAnalyzer 2100 (Agilent Technologies, Santa Clara, CA, USA). Two micrograms of RNA was reverse-transcribed into cDNA using SuperScript®III reverse transcriptase (Invitrogen, Carlsbad, CA, USA), and aliquots were stored at −20°C. Quantitative real-time TaqMan RT-PCR technology (7500Fast, Applied Biosystems, Foster City, CA, USA) was used to determine the expression levels of the selected target genes. The cycling conditions included a denaturing step at 95°C for 10 min and 50 cycles of 95°C for 15 s and 60°C for 1 min. The following TaqMan probes were used in the Q-RT-PCR analysis: rhodopsin, Hs00892431_m1; filaggrin, Hs00856927_g1; KRT1, Hs00196158_m1; KRT10, Hs01043110_g1; TGM3, Hs00162752_m1; KRT5, Hs00361185_m1. RPL13A, Hs04194366_g1 (Applied Biosystems, Foster City, CA, USA) was also amplified to normalize the variations in the cDNA levels across the different samples.

### Western Blot Analysis

NHEKs were lysed in RIPA cell lysis buffer containing a protease inhibitor and phosphatase inhibitor cocktail (Sigma, St Louis, MO, USA). The lysate was then subjected to centrifugation at 15,000×*g* for 20 min, and the supernatant was used for the analysis. The protein concentration was determined using the Bradford method with bovine serum albumin as the standard. All of the proteins (40 µg/well) were loaded and fractionated using SDS-PAGE and transferred onto nitrocellulose membranes. The membranes were blocked with 5% non-fat skim milk in TBST (10 mM Tris-HCl [pH 8.0], 150 mM NaCl, 0.015% Tween-20) for 1 hr at room temperature and then probed overnight at 4°C with anti-rhodopsin (RET-P1) antibody (Abcam, Cambridge, UK), and anti-p-ERK, -ERK, -p-CREB, -CREB, -p-CaMKII and -CaMKII antibodies (Cell Signaling, Boston, MA, USA). All of the blots were washed 3 times with TBST and then probed with horseradish peroxidase-conjugated goat anti-mouse or -rabbit IgG secondary antibodies (Bio-Rad, Hercules, CA, USA) at room temperature for 1 hr. The membranes were developed using ECL solution (Amersham Pharmacia Biotech, Piscataway, NJ, USA).

### Immunocytochemistry

For immunostaining, NHEKs were fixed with 10% formaldehyde at RT for 10 min and then permeabilized with 0.1% Triton X-100 for an additional 10 min. The blocking and diluent solution consisted of PBST containing 10% normal goat serum (Jackson ImmunoResearch Laboratories, West Grove, PA, USA). The cells were blocked at RT for 30 min and incubated for 1.5 hr with anti-rhodopsin antibody (Abcam, Cambridge, UK), followed by incubation with an Alexa 488 secondary antibody (Invitrogen, Carlsbad, CA, USA) for 1.5 hr. The nuclei were stained with DAPI (Sigma). PBST washes were performed between each step.

### Statistical Analyses

Data were analyzed by Student’s t-test or one-way analysis of variance (ANOVA) with Bonferroni post-hoc test using SPSS 20 software (IBM, New York, NY, USA). All of the measurements were obtained from at least three independent experiments carried out in triplicate, and values are expressed as the mean ± SEM.

## Results

### Rhodopsin is Expressed on the Plasma Membrane in NHEKs

Several previous reports have demonstrated that rhodopsin-like protein and SW-opsin are expressed in the upper layer of the epidermis, whereas LW-opsin is localized in the basal layer of the epidermis [21], [26]. To demonstrate that rhodopsin is expressed in monolayer-cultured NHEKs, we performed Western blot analysis and immunocytochemical staining using anti-rhodopsin antibodies. Western blot analysis revealed that rhodopsin was endogenously expressed as a monomer (∼37 kDa) and a dimer (∼75 kDa). The same band pattern was also shown in cells transfected with an expression vector overexpressing rhodopsin (Figure 1A). Immunocytochemical staining also showed that rhodopsin was localized at the plasma membrane (Figure 1B), similar to G protein-coupled receptors (GPCRs) [27]–[29].

### UVA and Violet Light (410 nm) can Increase Rhodopsin mRNA Expression and Decrease Keratin-10 (K10) and Keratin-1 (K1) mRNA Expression

**Figure 2:**
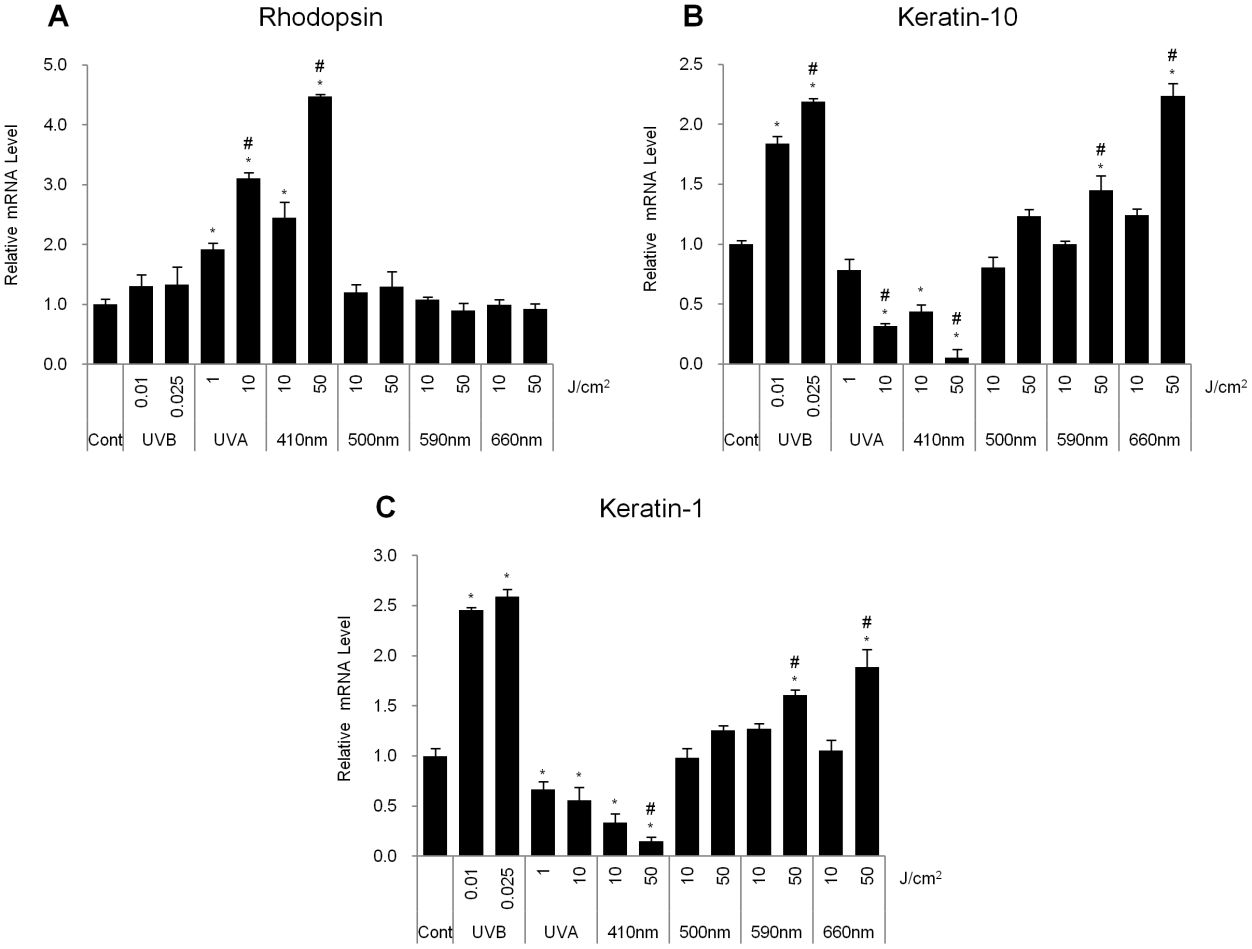
UVA and violet light irradiation induced rhodopsin expression and reduced the expression levels of differentiation markers in NHEKs. Using Q-RT-PCR analysis, the mRNA levels of rhodopsin and the keratinocyte differentiation markers K10 and K1 were measured. (A) The relative rhodopsin mRNA expression level. (B) Relative keratin-10 mRNA expression level. (C) Relative keratin-1 mRNA expression level was shown after exposure to UVB radiation (0.01, 0.025 J/cm^2^), UVA radiation (1, 10 J/cm^2^), or 410, 500, 590 or 660 nm wavelength light (10, 50 J/cm^2^). The values represent the mean ± SEM of the mRNA expression corresponding to various genes normalized to human RPL13A expression (n = 3 independent cell lines in triplicate). *p<0.05 versus the control. #p<0.05 versus lower irradiation dose of same wavelength light (One-way ANOVA with Bonferroni post-hoc test).

To investigate the effects of light irradiation of various wavelengths on the mRNA expression levels of rhodopsin in NHEKs, cells were irradiated with UVB, UVA (350–390 nm), or visible light (410, 500, 590, or 660 nm) (Figure S1). Unlike UVB, UVA increased the rhodopsin mRNA level (Figure 2A). Irradiation at the visible wavelength of 410 nm also significantly enhanced the rhodopsin mRNA level.

Previously, it has been reported that UVB radiation has acute effects on the expression of keratinocyte differentiation markers [4]. Q-RT-PCR analysis revealed that UVB showed an increase of approximately 2-fold in the mRNA expression levels of K10 and K1, which are representative markers of the spinous layer (Figures 2B and 2C). Interestingly, we found that the mRNA levels of K1 and K10 were significantly reduced in cells that had been exposed to UVA radiation and visible light at 410 nm (Figures 2B and 2C). In contrast, treatment at wavelengths of 590 and 660 nm but not 500 nm significantly increased the mRNA levels of K10 and K1 at 50 J/cm^2^ (Figures 2B and 2C).

### UVA or Violet Light Reduces the mRNA Levels of Specific Keratinocyte Differentiation Markers via Up-regulation of Rhodopsin

To investigate the relationship between rhodopsin and keratinocyte differentiation markers, small interfering RNA (siRNA)-mediated rhodopsin knockdown experiments were performed.

**Figure 3:**
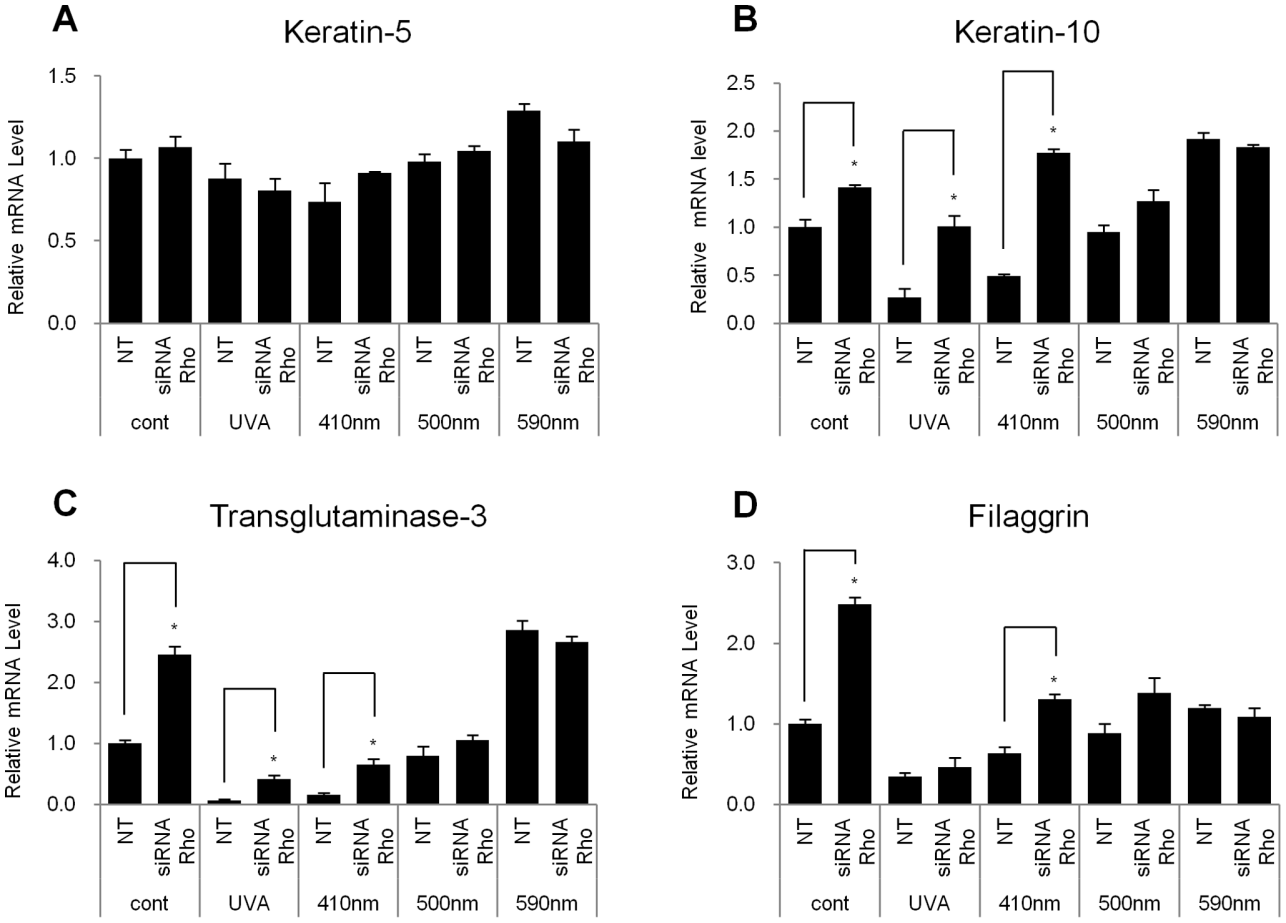
Knockdown of rhodopsin increased the expression levels of specific differentiation markers in NHEKs with or without irradiation of UVA or violet light. NHEKs were transfected with siRNA against rhodopsin (Rho) or scrambled siRNA and were irradiated with UVA (10 J/cm^2^), or 410 nm, 500 nm, 590 nm wavelength light (each 50 J/cm^2^). Q-RT-PCR was performed 24 hr after irradiation. All of the data are expressed as the relative mRNA level of (A) keratin-5, (B) keratin-10 (C) transglutaminase-3, and (D) filaggrin compared with control. The values represent the mean ± SEM of the mRNA expression corresponding to various genes normalized to human RPL13A expression (n = 3 independent cell lines in triplicate). *p<0.05.

We found that the knockdown of rhodopsin significantly increased the mRNA levels of specific differentiation markers K10, TGM3 (transglutaminase-3) and filaggrin but not K5 in the absence of any light irradiation (Figures 3 and S2). In addition, we observed that the introduction of rhodopsin-directed siRNA significantly restored the decreased K10 and TGM3 mRNA levels under UVA or 410 nm light irradiation suggesting that rhodopsin may mediate the cellular responses of UVA or 410 nm light radiation in NHEKs. However, the knockdown of rhodopsin did not induce significant change in the mRNA levels of the differentiation markers in NHEKs irradiated with 500 or 590 nm light (Figure 3).

**Figure 4:**
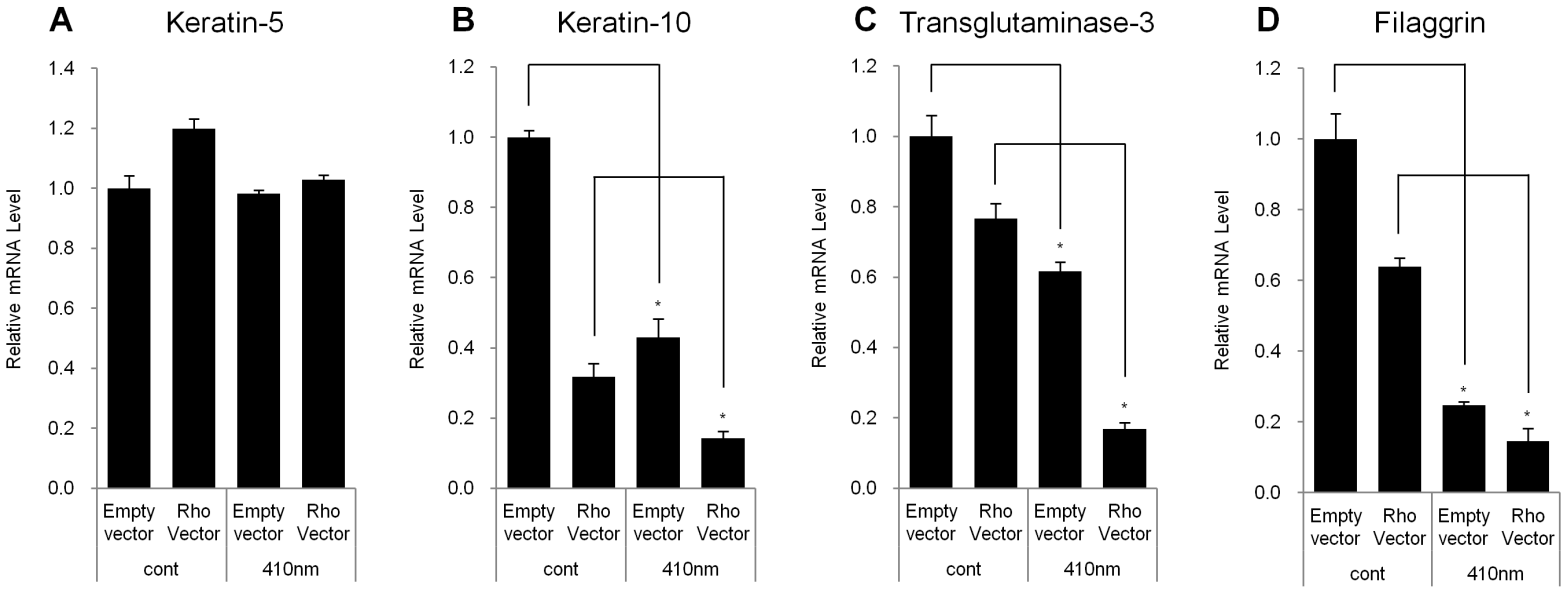
Over-expression of rhodopsin decreased the expression levels of differentiation markers in NHEKs. A pcDNA3.1 plasmid-encoding rhodopsin, or the empty pcDNA3.1 vector was transfected into NHEKs. After 48 hr, the transfected NHEKs were irradiated with UVA (10 J/cm^2^) or 410 nm wavelength light (50 J/cm^2^). After 24 hr, Q-RT-PCR was performed. All of the data are expressed as the relative mRNA level of (A) keratin-5, (B) keratin-10 (C) transglutaminase-3, and (D) filaggrin compared with control. The values represent the mean ± SEM of the mRNA expression corresponding to various genes normalized to human RPL13A expression (n = 3 independent cell lines in triplicate). *p<0.05.

Next, we examined whether exogenously over-expressed rhodopsin could influence the expression levels of differentiation markers in NHEKs. We observed that over-expressed rhodopsin did not have a deleterious effect on cell viability, independent of the light wavelengths (Figure S3). As expected, we found that the over-expression of rhodopsin resulted in a significant decrease in the mRNA expression levels of K10, TGM3 and filaggrin in the absence of light irradiation, although the mRNA expression level of K5 remained the same (Figure 4). Moreover, rhodopsin-over-expressed NHEKs exposed to violet light showed an additive effect compared to those that were not irradiated (Figure 4).

### Violet Light Decreases the Phosphorylation of cAMP Responsive Element-binding Protein (CREB)

**Figure 5:**
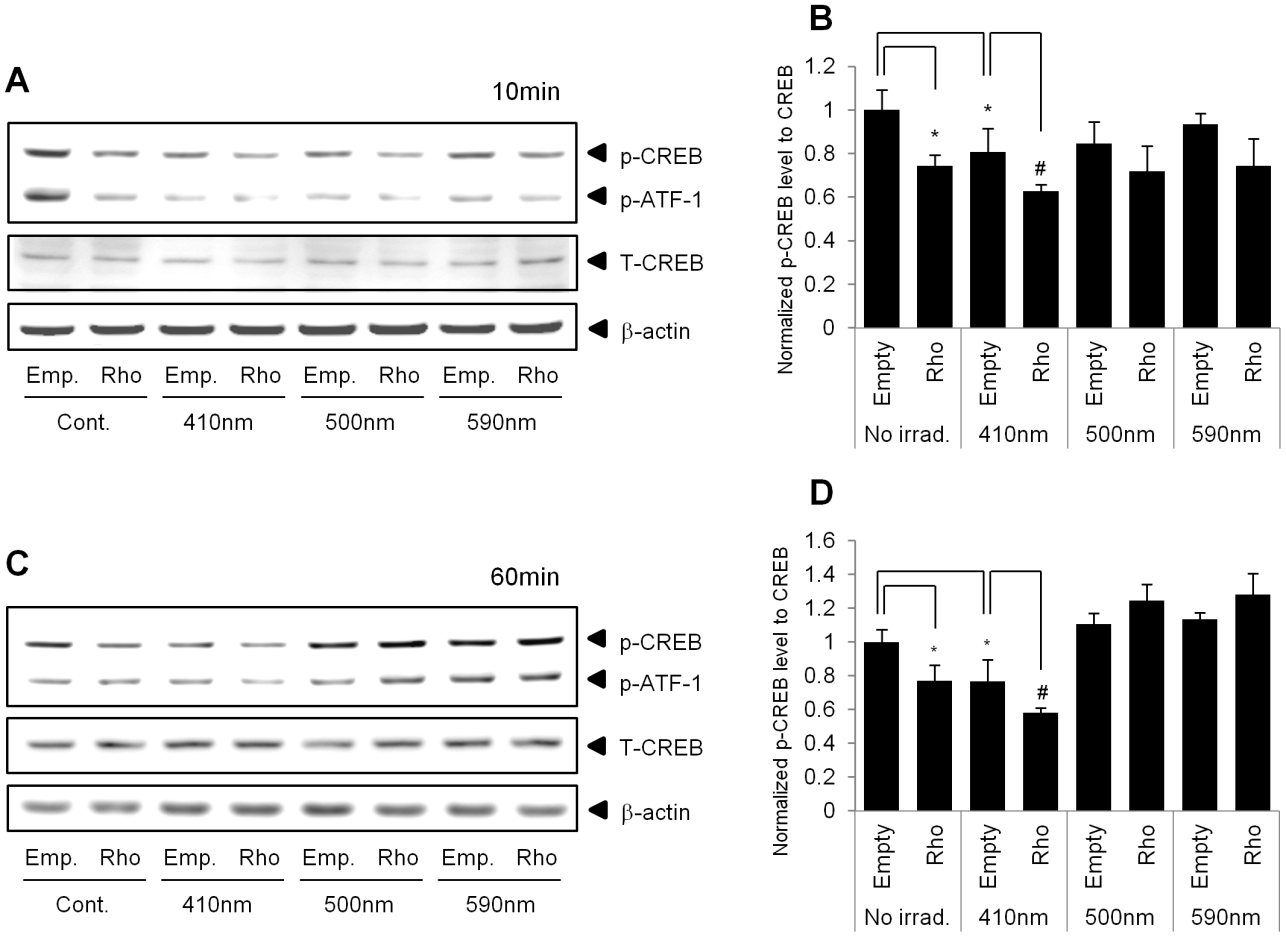
Phosphorylation levels of CREB (p-CREB) were decreased in rhodopsin-over-expressed NHEKs. NHEKs were transfected with a pcDNA3.1 empty vector (empty) or pcDNA3.1-rhodopsin plasmid (Rho). Next, the cells were irradiated with 410 nm, 500 nm, or 590 nm wavelength light for 10 min or 60 min. Cell lysates were analyzed by Western blot analysis. The representative images show the expression level of total CREB, p-CREB, and p-ATF-1 in NHEKs at 10 min (A) and 60 min (C). A statistical plot of the data displays the ratio of p-CREB/total CREB at 10 min (B) and 60 min (D), which is presented as the mean ± SEM (n = 3). *p<0.05 versus control empty vector and #p<0.05 versus 410 nm irradiated empty vector.

Rhodopsin is a G-protein-coupled receptor and utilizes the G protein transducin, (G_t_, a member of the G_i_ family). The G_i_ family includes G_αo_, G_αt_ and G_αz_, as well as G_αi_ (G_i_α subunit), and acts as an inhibitory signal for adenylyl cyclase, which can subsequently inhibit the cAMP–PKA signaling pathway [30]. cAMP responsive element-binding protein (CREB) is a downstream signal in G-protein-mediated signaling pathways. Thus, we measured the phosphorylation level of CREB after irradiation of cells with 410, 500, or 590 nm light in the absence or presence of exogenously over-expressed rhodopsin. Western blot analysis revealed that the over-expression of rhodopsin significantly decreased the phosphorylation level of CREB compared to that in empty vector-transfected cells (Figure 5). In addition, we found that irradiation with only 410 nm wavelength light, but not 500 or 590 nm wavelength light, significantly reduced the phosphorylation level of CREB, when the cells were exposed for 10 min (10 J/cm^2^) (Figures 5A and 5B) or 60 min (50 J/cm^2^) (Figures 5C and 5D). We observed that the phosphorylation level of CREB was decreased further in rhodopsin-over-expressed NHEKs after irradiation with 410 nm wavelength light than after no irradiation.

### Pertussis Toxin, a Gα_i_-protein Inhibitor, can Restore the Rhodopsin-induced Decrease in Specific Keratinocyte Differentiation Marker Levels

Pertussis toxin (PTX) can be isolated from the whooping cough-causing bacterium *Bordetella pertussis* and exhibits ADP-ribosyltransferase activity. PTX can effectively block cAMP fluctuations via the Gα_i_ protein pathway [30]. Thus, we used PTX as an inhibitor of the Gα_i_ protein to interfere with the violet light- and rhodopsin-mediated decrease in the mRNA expression levels of differentiation markers in NHEKs.

**Figure 6:**
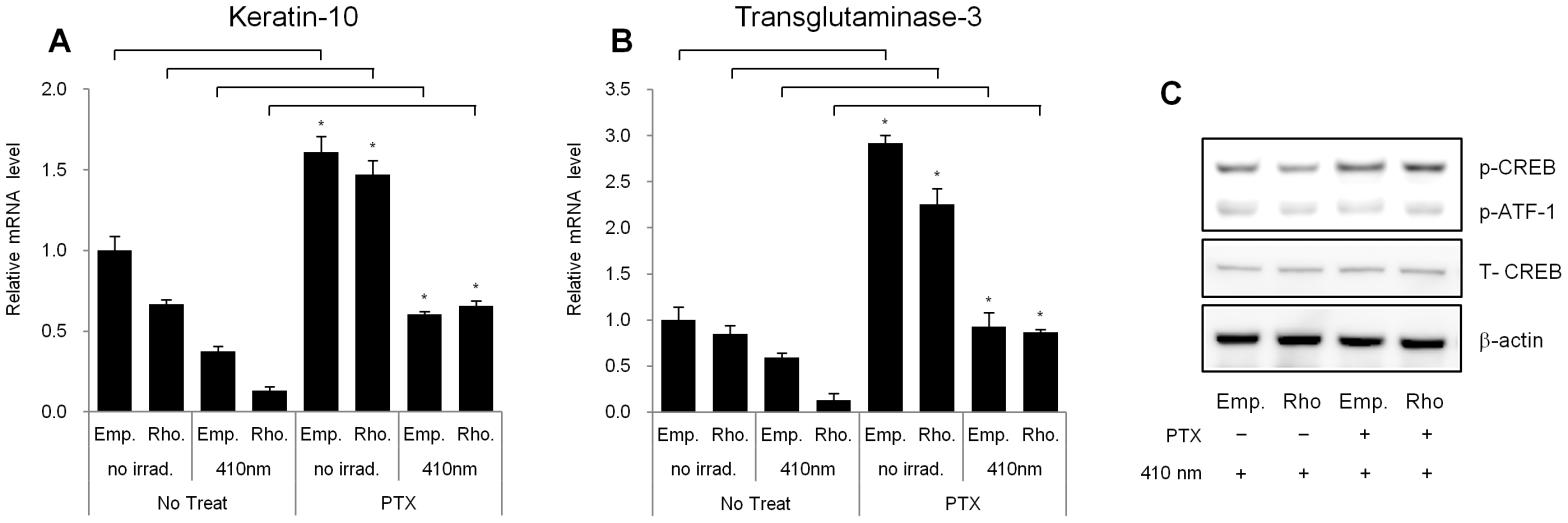
Pertussis toxin (PTX) restored the expression of differentiation markers in rhodopsin-over-expressed NHEKs. NHEKs were cultured and then stimulated with PTX pretreatment (100 ng/ml) for 1 hr prior to irradiation. The cells were incubated for 24 hr after irradiation with 410 nm wavelength light for 60 min. Q-RT-PCR was performed to assess the expression of (A) keratin-10 and (B) transglutaminase 3. The values represent the mean ± SEM of the mRNA expression corresponding to various genes relative to control which are normalized to human RPL13A expression (n = 3 independent cell lines in triplicate). *p<0.05 versus no treatment group. (C) Western blot analysis was also performed to detect the expression levels of total CREB, p-CREB, and p-ATF-1. The data are representative of at least three independent experiments.

Interestingly, treatment with PTX increased the mRNA expression levels of K10 and TGM3 (Figures 6A and 6B), Thus, it is possible that cAMP levels are up-regulated by PTX in NHEKs. We examined whether the reduced phosphorylation levels of CREB in rhodopsin-over-expressed NHEKs could be reversed by PTX treatment. We found that PTX treatment restored the reduced phosphorylation level of CREB in rhodopsin-over-expressed NHEKs after irradiation with 410 nm wavelength light (Figure 6C). These results suggested that Gα_i_ protein-related signaling is involved in the rhodopsin induced change of differentiation marker expression levels in NHEKs, similar to visual phototransduction in the human retina [28], [29].

### BAPTA-AM and W7 cannot Restore the Rhodopsin-induced Reduction in Specific Keratinocyte Differentiation Marker Levels

UVA irradiation can elicit retinal-dependent Ca^2+^ flux and mobilization via rhodopsin in HEMs [26]. To demonstrate whether transient Ca^2+^ flux through rhodopsin irradiated with 410 nm wavelength light has an effect on the expression of keratinocyte differentiation marker gene transcripts, we used an intracellular Ca^2+^ chelator, BAPTA-AM, and the Ca^2+^/CaM antagonist, W7.

**Figure 7:**
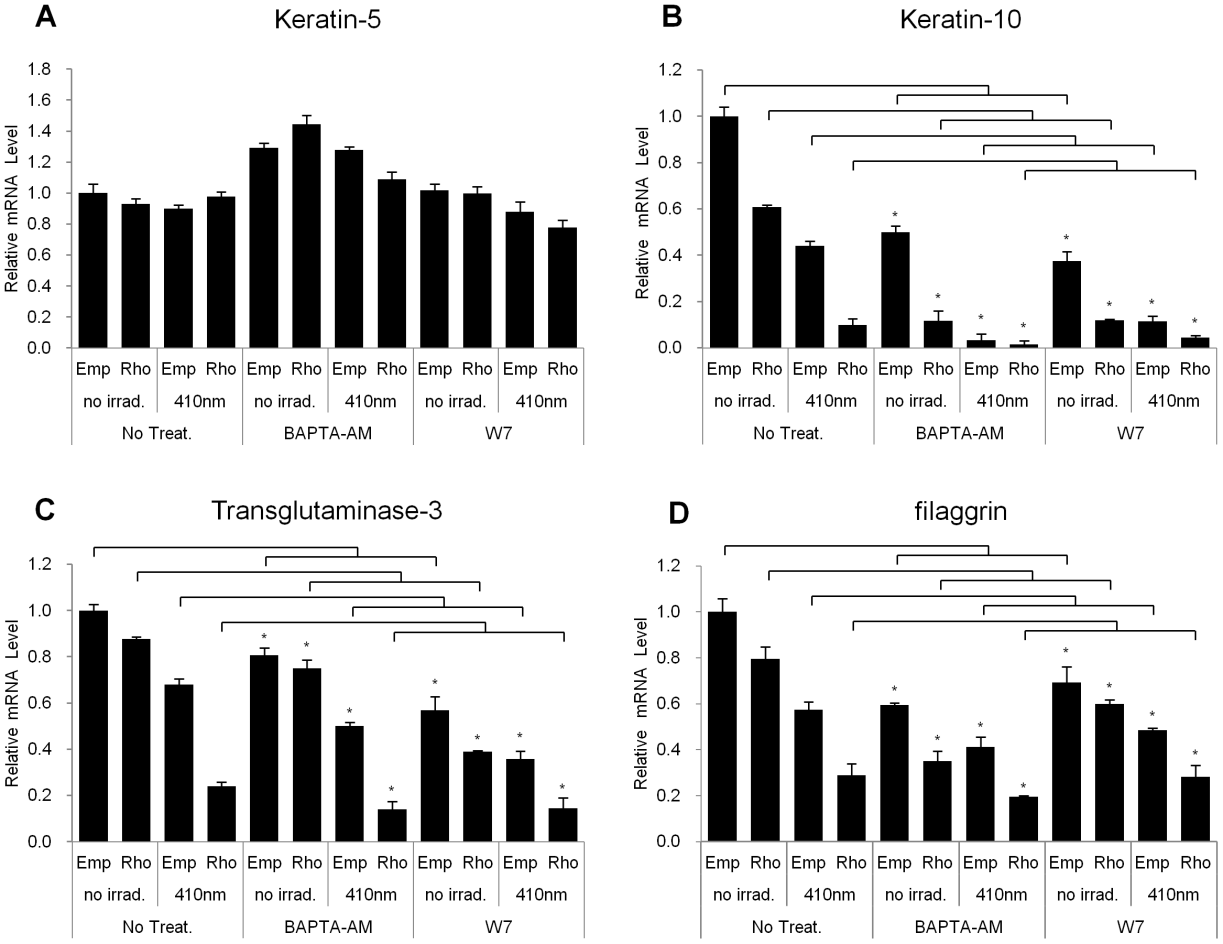
BAPTA-AM and W7 had no significant effects on the expression levels of the differentiation markers in rhodopsin-over-expressed NHEKs. NHEKs were cultured and then treated with BAPTA-AM (10 mM) or W7 (40 mM) 1 hr before irradiation. The cells were incubated for 24 hr after irradiation with 410 nm wavelength light for 60 min. Q-RT-PCR was performed to assess the expression of (A) keratin-5, (B) keratin-10, (C) transglutaminase-3, and (D) filaggrin. The data are representative of at least three independent experiments. The values represent the mean ± SEM of the mRNA expression corresponding to the various genes relative to control which are normalized to human RPL13A expression (n = 3 independent cell lines in triplicate) *p<0.05 versus no treatment group.

In the absence of irradiation at 410 nm, we found that mRNA levels of the differentiation markers, K10, TGM3, and filaggrin but not K5, were significantly reduced upon treatment with BAPTA-AM compared to control (Figure 7). In addition, we observed a strengthening of its effects when rhodopsin was over-expressed. These effects were even more prominent after irradiation with 410 nm light. We also found that W7 activity showed a pattern similar to those obtained with the BAPTA-AM treatment (Figure 7). These results indicated that transient Ca^2+^ flux via over-expressing rhodopsin or exposing cells to 410 nm wavelength light has exerted no significant influence on the expression of keratinocyte differentiation markers, although these two chemicals showed a tendency to down-regulate the differentiation markers.

## Discussion

Several previous reports suggested that there are photoreceptors sensing the visible lights in the cutaneous system similar to retinal system [7], [21], [26], [31]. Rod and cone photoreceptors-like proteins are expressed in human dermis, although the physiological effects of those proteins are not elucidated [21]. UVA induces melanin synthesis through activation of rhodopsin in human melanocytes [26]. In this study, we demonstrated that the rhodopsin-related phototransduction of violet light at 410 nm is involved in the down-regulated expression of differentiation markers in NHEKs. We sought to elucidate the physiological roles and signaling pathways of rhodopsin, a non-ocular opsin, in NHEKs. In rod photoreceptors, rhodopsin is present in monomeric and dimeric forms [32], [33]. We found that rhodopsin was also expressed and localized at the plasma membrane in NHEKs (Figure 1), suggesting that rhodopsin can act as a G protein receptor, similar to the photoreceptors involved in vision. In UVA- or violet light-irradiated NHEKs, rhodopsin mRNA levels were significantly increased, indicating that rhodopsin might have physiological roles in NHEKs (Figure 2A).

It is well-known that UVB radiation can increase the expression of differentiation markers, such as K10, K1, TGM3 and filaggrin, and exposing human skin cells to UVA, 419 nm, 426 nm or 453 nm wavelength light induces the up-regulation of differentiation markers after 3 exposures for 3 days (approximate accumulated dosage of 15–198 J/cm^2^) [9]. However, in this study, we found that UVA as well as violet light, although violet light was more effective than UVA, decreased the mRNA expression levels of differentiation markers in NHEKs (Figures 2B and 2C). We adopted a single exposure for our experiments to determine whether rhodopsin directly affects signaling pathways in NHEKs. Thus, we irradiated the cells only once and then analyzed the mRNA levels of differentiation markers within 24 hr of light exposure (10–50 J/cm^2^). This difference in the exposure number and duration might be the reason for the different expression patterns of keratinocyte differentiation markers.

We also demonstrated that the effects of violet light on the down-regulation of the expression of keratinocyte differentiation markers were mediated via the specific stimulation of rhodopsin. The rhodopsin-directed siRNA recovered the mRNA levels of the differentiation markers, K10, K1, and filaggrin, which were decreased in response to irradiation with violet light in NHEKs (Figure 3). In contrast, when rhodopsin was transiently over-expressed in NHEKs, the differentiation markers were significantly down-regulated, and the expression levels were further decreased after irradiation with violet light (Figure 4), indicating that rhodopsin suppressed the differentiation of NHEKs. We then sought to uncover the signaling pathways that underlie this rhodopsin-induced phenomenon. According to previous reports, photo-excited rhodopsin (R*) can activate the heterotrimeric transducin and cause a dissociation of its α-subunit. The dissociated α-subunit then activates cGMP phosphodiesterase, which breaks down cGMP, an intracellular second messenger, resulting in hyperpolarization in the retina [34], [35]. In addition, rhodopsin is also known to be involved in cAMP signaling cascades. It has been reported that light exposure causes a decrease in cAMP levels in rod cells [36], [37]. In this study, we had a difficulty to measure the lower values of cGMP and cAMP concentration than the basal levels of cGMP and cAMP when rhodopsin was up-regulated by over-expression or violet light irradiation. Thus, using indirect approach, we investigated whether the phosphorylation level of CREB, a representative downstream factor in cAMP signaling, was regulated by rhodopsin. In the absence of light irradiation, the over-expression of rhodopsin significantly reduced the phosphorylation level of CREB compared to that in empty vector-transfected NHEKs (Figure 5). To further elucidate the mechanisms mediating this phenomenon, we utilized PTX as an inhibitor of G_i_ proteins. Interestingly, the decreased p-CREB levels caused by the over-expression of rhodopsin were restored with PTX treatment (Figure 6), suggesting that violet light phototransduction via rhodopsin is associated with the G_i_ protein signaling pathway. Furthermore, we found that ERK phosphorylation was increased with the over-expression of rhodopsin and/or with exposure to violet light (Figure S4), suggesting that this process might involve the G_i_-ßγ protein, which is known to activate ERK [38], [39]. However, we did not examine whether p-ERK levels decrease in response to PTX treatment, because PTX can directly activate ERK phosphorylation, which is involved in the PKC-dependent signaling pathway [39]. We also analyzed the levels of p-STAT in the c-Src signaling pathway via Gα_i_ proteins and the levels of p-CaMKII induced by Ca^2+^ modulation within 60 min. However, we were unable to observe any changes in the phosphorylation levels in violet light-irradiated or rhodopsin over-expressed NHEKs (data not shown). The use of W7 and BAPTA-AM to chelate Ca^2+^ or inhibit the effects of Ca^2+^-related signaling, demonstrated a tendency to down-regulate the expression of keratinocyte differentiation markers, although these drugs did not affect the activity of rhodopsin. In addition, UVA or violet light irradiation did not evoke Ca^2+^ transients independent of the over-expression of rhodopsin and treatment with 9 or 11-cis-retinal (data not shown). Thus, it is plausible that the rhodopsin-induced reduction in the expression of keratinocyte differentiation markers was not significantly affected by Ca^2+^ as a second messenger. Rhodopsin chromophores such as 9-cis, 11-cis-retinal or all-trans-retinal may markedly reduce the level of differentiation marker gene transcripts. Our HPLC analysis revealed that neither vitamin A nor other retinal derivatives were found in the culture medium (data not shown). Thus, it still remains to be determined whether NHEKs intrinsically contain vitamin A or other retinal derivatives.

According to previous reports, red light accelerated the recovery of epidermal barrier, whereas blue light delayed it. In contrast, green light had no effect on the epidermal barrier recovery [9], [12]. We also observed that violet light reduced differentiation of NHEKs. These results suggest that a distinct wavelength can be clinically used for treating abnormal skin disorders such as hyper-differentiation and proliferation-related skin diseases.

In summary, we demonstrated that violet light down-regulates specific differentiation markers of NHEKs via non-ocular rhodopsin. We suggest that this phenomenon might be mediated by the down-regulation of CREB phosphorylation via the Gα_i_ and cAMP signaling pathways.

## Supporting Information

Figure S1 **UVA and visible light-emitting diode (LED) spectra and temperature after irradiation.** (A) The wavelength spectra for UVA, 410 nm, 500 nm, 590 nm and 660 nm wavelength light of the irradiation system. This system has a narrow wavelength range and a high-energy irradiance power (for details, please refer to the Materials and Methods). (B) Effect of each light irradiation on the temperature of culture medium.
(TIF)

Figure S2 **Knockdown of** **Rhodopsin in NHEKs.** NHEKs were transfected with siRNA against rhodopsin or scrambled siRNA and were exposed to UVA radiation (10 J/cm^2^) or to 410 nm, 500 nm, or 590 nm wavelength light (each 50 J/cm^2^). Q-RT-PCR was performed 24 hr after irradiation. All of the data are expressed relative to the control mRNA level of rhodopsin. The values represent the mean ± SEM of the mRNA expression of rhodopsin normalized to human RPL13A expression (n = 3 independent cell lines in triplicate). *p<0.05 versus NT (non-targeting siRNA) vector.
(TIF)

Figure S3 **Effects of over-expressing rhodopsin combined with light irradiation on the viability of NHEKs.** NHEKs were exposed to various radiation wavelengths after transfection. 24 hr after light irradiation, the percentage of viable cells was assessed using the CCK-8 assay (n = 3 independent cell lines in triplicate).
(TIF)

Figure S4 **Phosphorylation levels of ERK in rhodopsin-over-expressed NHEKs.** After exposure to violet light for 60 min, the phosphorylation levels of ERK were detected in NHEKs over-expressing rhodopsin (Rho) and control empty vector (Empty) by using Western blot analysis. ß-actin was used as a loading control. The images are representative of three independent experiments.
(TIF)
