# Supplementary figures and images for: Violet Light Down-Regulates the Expression of Specific Differentiation Markers through Rhodopsin in Normal Human Epidermal Keratinocytes
Source: PLoS One. 2013 Sep 17;8(9):e73678. doi: 10.1371/journal.pone.0073678 (PMC3775733; doi:10.1371/journal.pone.0073678)

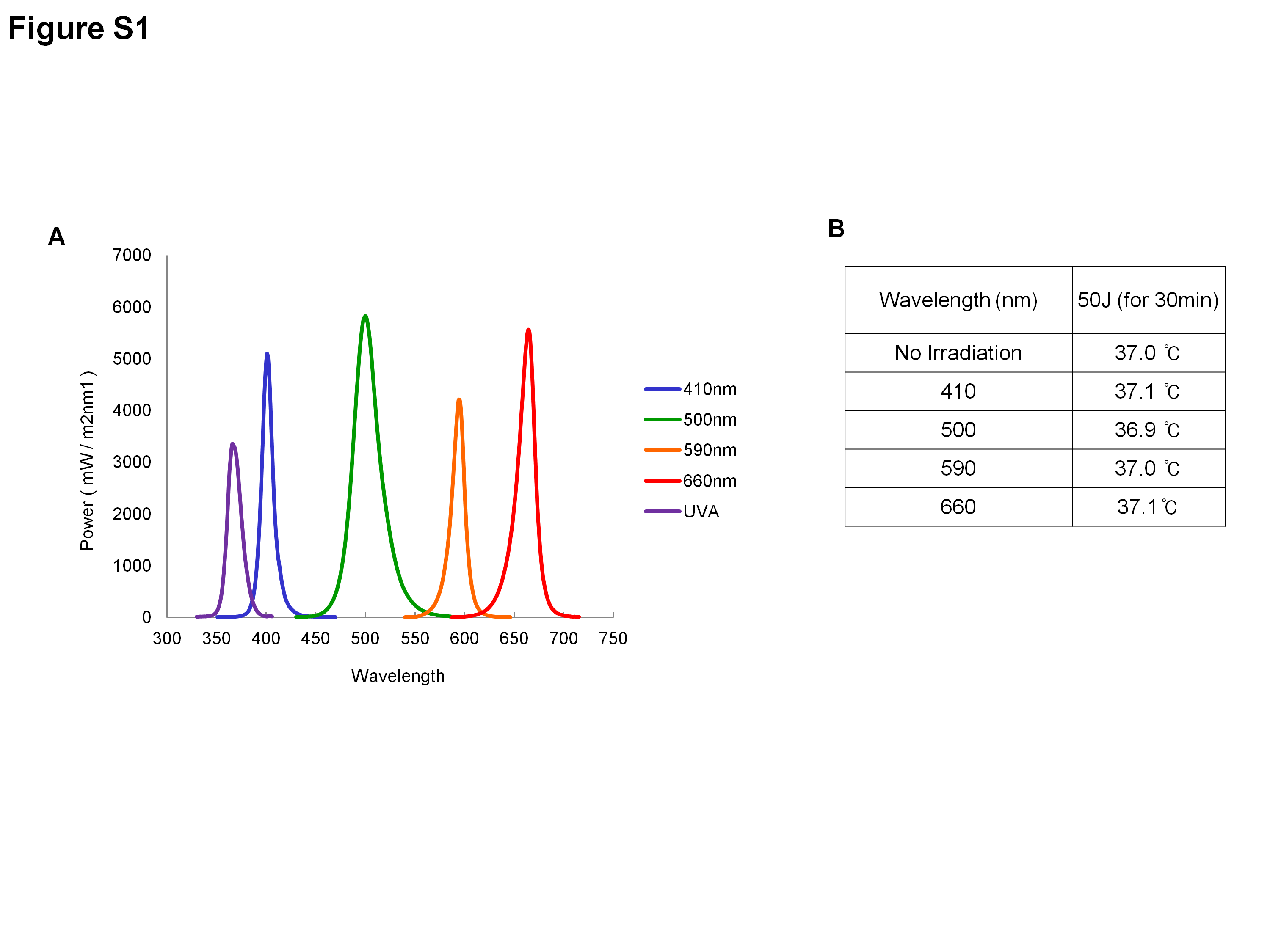

Supplement: Figure S1 — UVA and visible light-emitting diode (LED) spectra and temperature after irradiation. (A) The wavelength spectra for UVA, 410 nm, 500 nm, 590 nm and 660 nm wavelength light of the irradiation system. This system has a narrow wavelength range and a high-energy irradiance power (for details, please refer to the Materials and Methods). (B) Effect of each light irradiation on the temperature of culture medium. (TIF) [file pone.0073678.s001.tif]

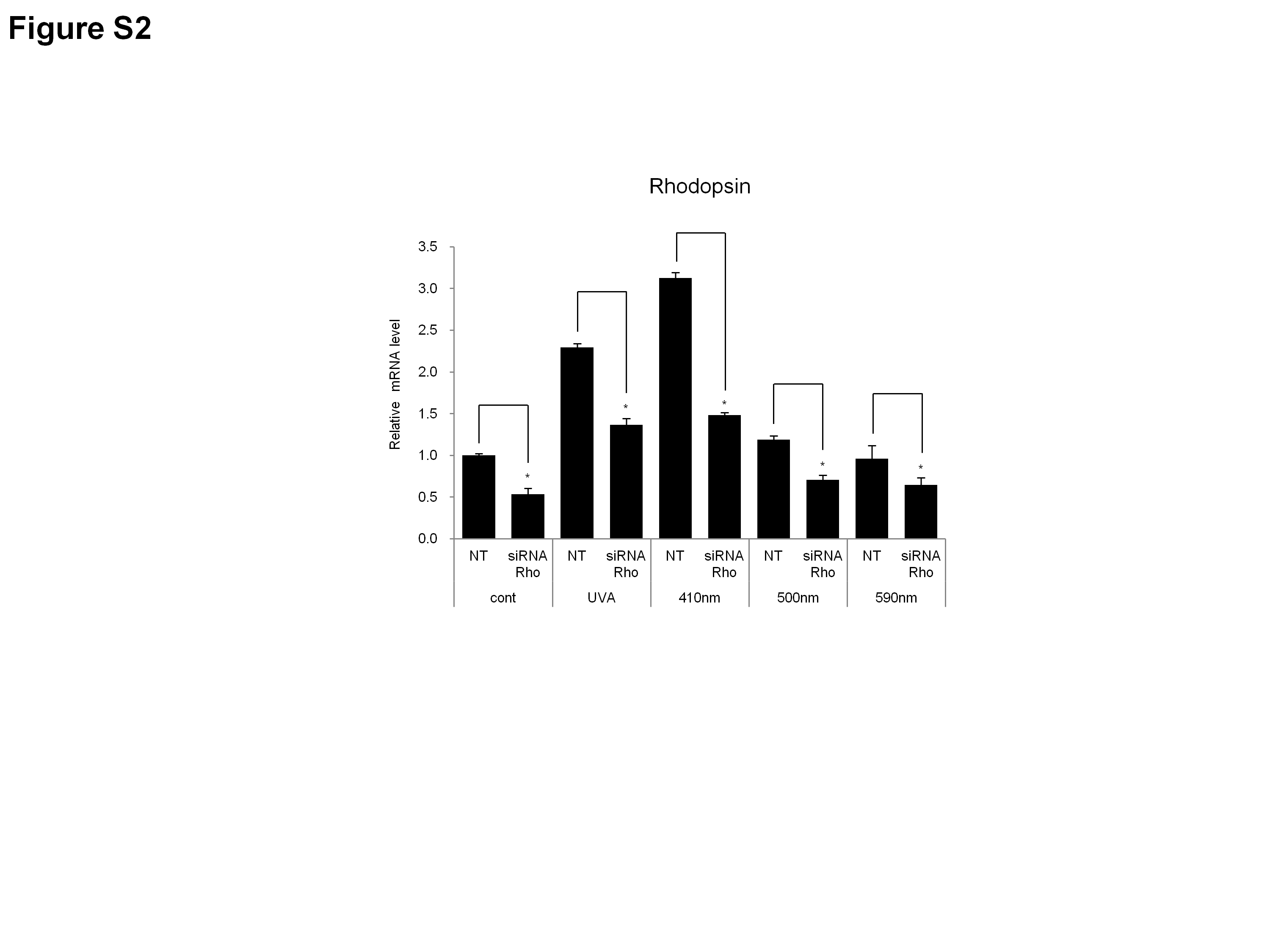

Supplement: Figure S2 — Knockdown of Rhodopsin in NHEKs. NHEKs were transfected with siRNA against rhodopsin or scrambled siRNA and were exposed to UVA radiation (10 J/cm2) or to 410 nm, 500 nm, or 590 nm wavelength light (each 50 J/cm2). Q-RT-PCR was performed 24 hr after irradiation. All of the data are expressed relative to the control mRNA level of rhodopsin. The values represent the mean ± SEM of the mRNA expression of rhodopsin normalized to human RPL13A expression (n = 3 independent cell lines in triplicate). *p<0.05 versus NT (non-targeting siRNA) vector. (TIF) [file pone.0073678.s002.tif]

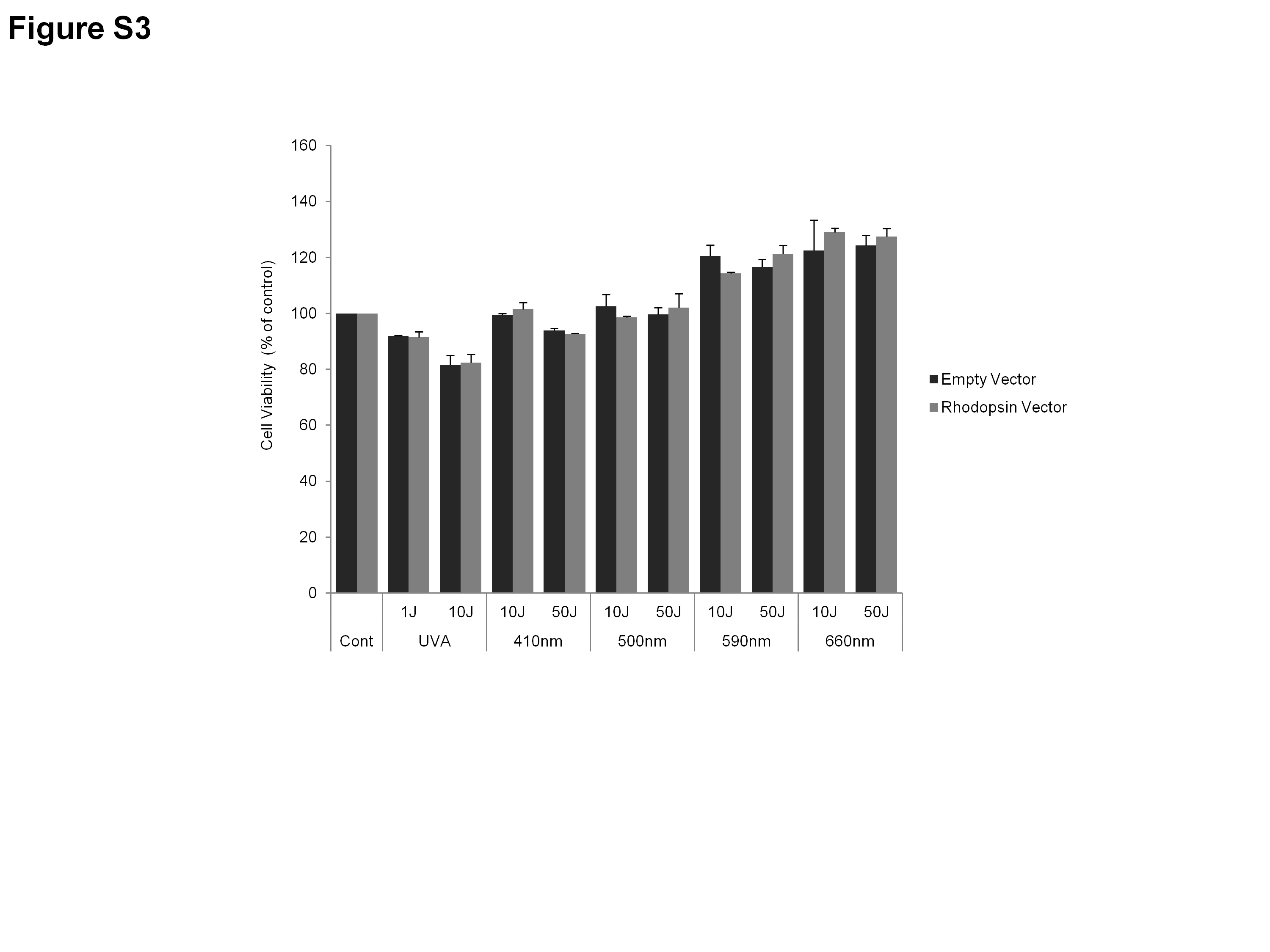

Supplement: Figure S3 — Effects of over-expressing rhodopsin combined with light irradiation on the viability of NHEKs. NHEKs were exposed to various radiation wavelengths after transfection. 24 hr after light irradiation, the percentage of viable cells was assessed using the CCK-8 assay (n = 3 independent cell lines in triplicate). (TIF) [file pone.0073678.s003.tif]

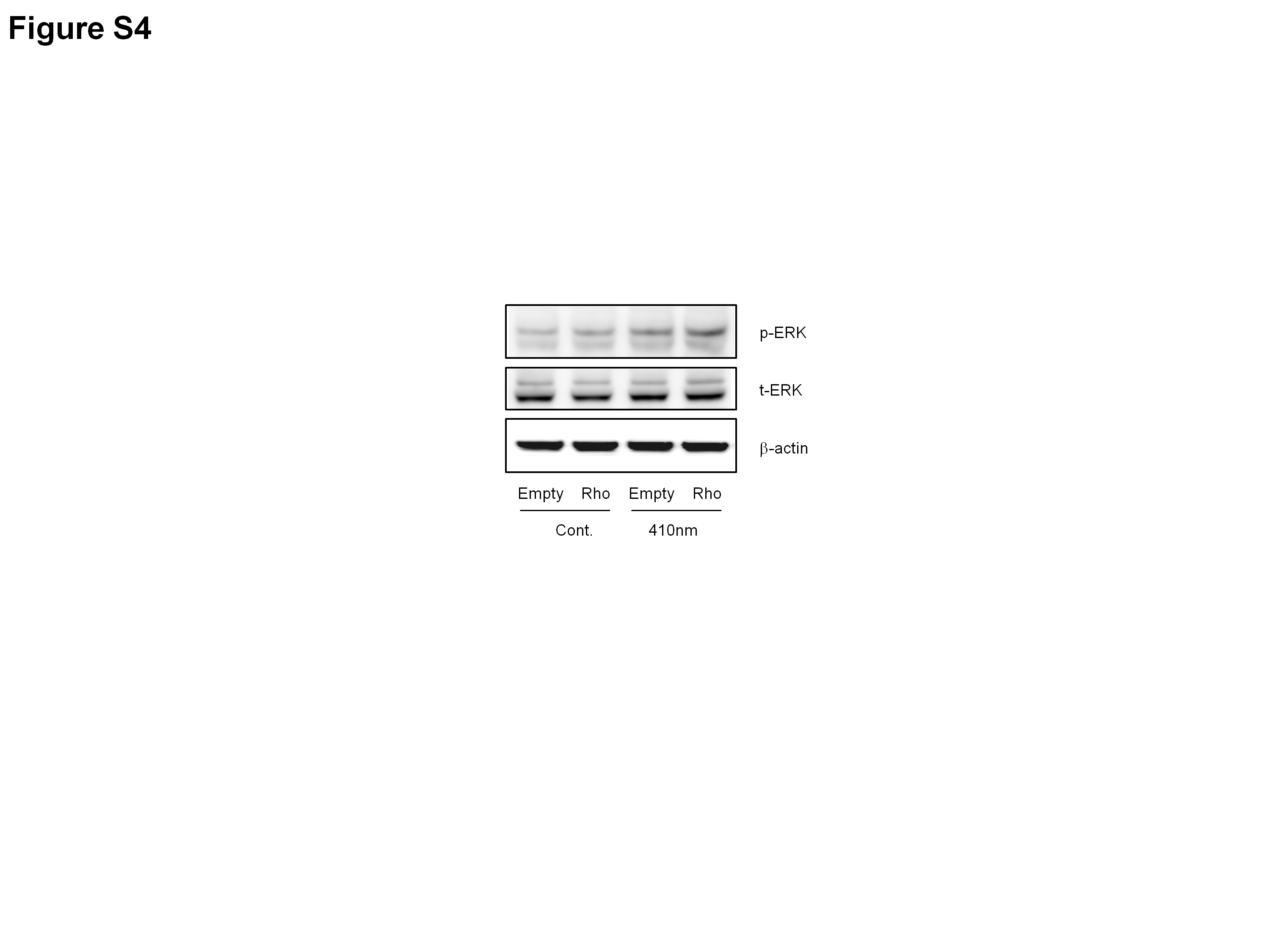

Supplement: Figure S4 — Phosphorylation levels of ERK in rhodopsin-over-expressed NHEKs. After exposure to violet light for 60 min, the phosphorylation levels of ERK were detected in NHEKs over-expressing rhodopsin (Rho) and control empty vector (Empty) by using Western blot analysis. ß-actin was used as a loading control. The images are representative of three independent experiments. (TIF) [file pone.0073678.s004.tif]
